# Severe Pancytopenia Secondary to Zinc Toxicity from Age-Related Eye Disease Study Vitamin Supplementation

**DOI:** 10.1177/24741264261436350

**Published:** 2026-03-29

**Authors:** Rui Wang, Alia K. Durrani

**Affiliations:** 1 The Retina Institute of St. Louis, St. Louis, MO, USA

**Keywords:** age-related macular degeneration, Age-Related Eye Disease Study, zinc toxicity, hypocupremia, copper deficiency, pancytopenia

## Abstract

**Purpose:** To report a case of zinc-induced hypocupremia associated with Age-Related Eye Disease Study 2 supplementation presenting with pancytopenia. **Methods:** A single case was retrospectively reviewed. **Results:** An 86-year-old woman taking Age-Related Eye Disease Study 2 supplementation presented to a hematology–oncology clinic with severe fatigue and pancytopenia. Laboratory evaluation demonstrated elevated zinc levels and low copper levels consistent with zinc-induced hypocupremia. Age-Related Eye Disease Study supplementation was discontinued, and the patient was started on copper supplementation. At the 4-month follow-up, the patient demonstrated near normalization of hematologic parameters and resolution of symptoms. **Conclusions:** Zinc-induced hypocupremia is a rare but potentially fatal complication of Age-Related Eye Disease Study 2 supplementation and may present with pancytopenia. Clinicians should consider this diagnosis in patients taking zinc-containing supplements who present with unexplained cytopenias.

## Introduction

Age-related macular degeneration (AMD) is a leading cause of blindness in developed countries and is estimated to affect approximately 200 million people worldwide.^1,2^ Treatment options for early and intermediate AMD remain limited and generally revolve around lifestyle modification and risk factor control.^3^ The Age-Related Eye Disease Study demonstrated that daily oral supplementation with antioxidant vitamins and minerals reduced the risk of progression to advanced AMD by 25% over 5 years.^4^ The Age-Related Eye Disease Study formulation included vitamin C 500 mg, vitamin E 40 IU, beta-carotene 15 mg, copper 2 mg, and zinc 80 mg. Subsequently, the Age-Related Eye Disease Study 2 formulation was found to be equally efficacious and replaced beta-carotene with lutein and zeaxanthin, as beta-carotene was associated with a higher incidence of lung cancer in former smokers.^5^ Herein, we describe a unique case of severe pancytopenia resulting from zinc-induced hypocupremia associated with Age-Related Eye Disease Study 2 supplementation.

## Case Report

An 86-year-old woman with a history of exudative AMD reported a recent history of progressive fatigue over 6 to 12 weeks to her primary care provider. Initial evaluation revealed severe pancytopenia, and the patient was referred to the hematology–oncology clinic for further assessment. Her medical history was notable for carotid and peripheral vascular disease, for which she had undergone carotid endarterectomy 4 years prior, superior mesenteric artery stenosis treated with stent placement 3 years ago, osteoporosis, and degenerative disc disease.

The patient lived independently, reported consuming no more than 1 alcoholic drink a day, and had a 40 pack-year smoking history, having quit 30 years earlier. She denied allergies or food intolerances and reported consuming a Western-pattern diet. Review of systems was notable for numbness and a tingling sensation in both feet and recent worsening of chronic back pain. The patient denied recent infections, hospitalizations, new medications, weight loss, night sweats, or fevers. Her medications included aspirin 81 mg daily, clopidogrel 75 mg daily, pregabalin 75 mg as needed, tramadol 50 mg as needed, Age-Related Eye Disease Study 2 supplementation twice daily, and denosumab 60 mg subcutaneously every 6 months. She reported a 2-year history of Age-Related Eye Disease Study vitamin supplementation.

On physical examination, the patient was afebrile, normotensive, and had a pulse of 80 beats per minute. The hematologist noted senile purpura-like findings on both upper extremities, but the remainder of the examination was unremarkable. Laboratory testing demonstrated severe pancytopenia, with hemoglobin of 8.4 g/dL (normal: 12.0–15.5 g/dL), white blood count of 2.2 × 10^3^/µL (normal: 4.5–11 × 10^3^/µL) with an absolute neutrophil count of 500/µL (normal: 2500– 7000/µL), and a platelet count of 91 × 10^3^/µL (normal: 150–450 × 10^3^/µL). Notably, the patient’s complete blood count 1 year prior was unremarkable.

Given the severity of the pancytopenia and the absence of an obvious clinical cause, further evaluation was recommended, including a computed tomography (CT) scan, additional laboratory evaluation, and a bone marrow biopsy.

CT of the abdomen and pelvis demonstrated no lymphadenopathy and/or splenomegaly. A bone marrow biopsy of the left posterior iliac crest demonstrated mild hypercellularity for age (40%; normal: 20%—40%), absent storage iron, and no ringed sideroblasts. Fluorescence in situ hybridization panel was negative for myelodysplastic syndrome.

Additional laboratory evaluation was notable for a serum copper level <10 µg/dL (lower limit of normal: 70 µg/dL), ceruloplasmin <3 µg/dL (lower limit of normal: 16 µg/dL), serum zinc level of 211 µg/dL (upper limit of normal: 116 µg/dL), and serum iron level of 20 µg/dL (normal: 60–160 µg/dL). These findings were consistent with copper deficiency secondary to zinc toxicity. The patient was advised to discontinue Age-Related Eye Disease Study 2 supplementation due to its high zinc content and was started on oral copper supplementation at 9 mg daily.

The patient was evaluated 4 weeks after initiation of oral copper supplementation and reported no change in symptoms. However, repeat complete blood count demonstrated improvement, with a hemoglobin level of 9.7 g/dL, platelet count of 163 × 10^3^/µL, and a white blood cell count of 7.1 × 10^3^/µL. The patient was instructed to continue oral copper supplementation and was reevaluated 3 months later, at which time she reported significant improvement in energy and resolution of tingling in both her feet. Repeat complete blood count demonstrated persistent but improving anemia (hemoglobin 11.0 g/dL) with normalized platelet count (172 × 10^3^/µL) and white blood count (7.3 × 10^3^/µL). Follow-up serum zinc and copper levels were within normal limits.

## Conclusions

Zinc is an essential trace mineral that plays an important role in numerous physiologic processes, including immune function, metabolism, and tissue regeneration.^6^ Among adults aged over 71 years, approximately 37% of the United States population reports taking zinc supplements.^7^ The recommended dietary allowance for zinc is 8 mg and 11 mg per day for women and men, respectively, while the tolerable upper intake level is 40 mg per day.^8^ The Age-Related Eye Disease Study evaluated a formulation containing high doses of antioxidants and minerals, including 80 mg of zinc oxide. The subsequent Age-Related Eye Disease Study 2 investigated whether reducing the zinc oxide dose to 25 mg would affect efficacy and found that the lower dose did not significantly affect the effectiveness of the formulation. However, the Age-Related Eye Disease Study 2 ultimately retained the 80 mg zinc content because the original Age-Related Eye Disease Study was the only placebo-controlled trial evaluating the formulation.^4,5^

Although zinc intake has many beneficial properties, excess accumulation can cause serious adverse effects. Given that zinc and copper are divalent cations that compete within the gastrointestinal tract, excess zinc intake interferes with copper absorption.^9^ Hypocupremia, or copper deficiency, can lead to nonspecific presentations affecting the hematologic, neurologic, immunologic, dermatologic, cardiovascular, and skeletal systems. Among these, neurologic and hematologic abnormalities are considered the most severe manifestations.^10^ Neurologic deficits may range from mild sensory symptoms, such as a tingling sensation of the extremities, to severe ambulatory dysfunction. Anemia and leukopenia are the most common hematologic presentations, while pancytopenia, as observed in our patient, is considered a rare manifestation of hypocupremia.^10^

The patient had been taking supplements containing the Age-Related Eye Disease Study 2 formulation for approximately 2 years before presenting with severe fatigue. This timeline is consistent with prior reports demonstrating an average delay of approximately 12 months between symptom onset and diagnosis of hypocupremia in symptomatic patients.^10^ The patient denied additional vitamin supplementation and reported a typical Western diet. Notably, she had a history of superior mesenteric artery ischemia, which may have impaired nutrient absorption in the proximal small intestine and increased her susceptibility to hypocupremia. Upon discontinuation of the Age-Related Eye Disease Study 2 supplementation and initiation of oral copper supplementation, the patient experienced significant improvement in her symptoms and near normalization of her pancytopenia 4 months after diagnosis.

Serum copper levels typically begin to improve within 3 to 4 weeks, and copper supplementation is generally recommended for 1 to 6 months, depending upon the dose of oral copper supplementation and the severity of deficiency.^10^

In conclusion, this report describes a rare case of zinc toxicity associated with Age-Related Eye Disease Study 2 supplement use. To our knowledge, this is the first case of Age-Related Eye Disease Study 2 supplementation leading to zinc toxicity in the English ophthalmologic literature. Although zinc toxicity is exceedingly rare, this case highlights the importance of recognizing symptoms and relevant risk factors for zinc-induced hypocupremia in patients taking Age-Related Eye Disease Study 2 supplements. Provider knowledge and appropriate patient counseling remain critical.

In addition, some patients may be taking multiple vitamin supplements concurrently, unknowingly resulting in cumulative zinc intake exceeding that contained in the Age-Related Eye Disease Study 2 formulation. Early diagnosis and treatment, including cessation of zinc-containing supplements and prompt initiation of copper supplementation, may help prevent irreversible neurologic complications.
